# Role of Keap1-Nrf2 signaling in depression and dietary intake of glucoraphanin confers stress resilience in mice

**DOI:** 10.1038/srep30659

**Published:** 2016-07-29

**Authors:** Wei Yao, Ji-chun Zhang, Tamaki Ishima, Chao Dong, Chun Yang, Qian Ren, Min Ma, Mei Han, Jin Wu, Hiroyuki Suganuma, Yusuke Ushida, Masayuki Yamamoto, Kenji Hashimoto

**Affiliations:** 1 Division of Clinical Neuroscience, Chiba University Center for Forensic Mental Health, Chiba 260-8670, Japan; 2 Innovation Division, KAGOME CO. LTD., Nasushiobara, Tochigi 329-2762, Japan; 3 Departments of Medical Biochemistry and Respiratory Medicine, Tohoku University Graduate School of Medicine, Sendai 980-8575, Miyagi, Japan

## Abstract

The transcription factor Keap1-Nrf2 system plays a key role in inflammation which is involved in depression. We found lower expression of Keap1 and Nrf2 proteins in the prefrontal cortex (PFC), CA3 and dentate gyrus (DG) of hippocampus in mice with depression-like phenotype compared to control mice. Serum levels of pro-inflammatory cytokines in *Nrf2* knock-out (KO) mice were higher than those of wild-type mice, suggestive of enhanced inflammation in KO mice. Decreased brain-derived neurotrophic factor (BDNF) and its receptor tropomyosin-receptor-kinase B (TrkB) signaling in the PFC, CA3 and DG plays a role in the depression-like phenotype of *Nrf2* KO mice. TrkB agonist 7,8-dihydroxyflavone, but not antagonist ANA-12, produced antidepressant effects in *Nrf2* KO mice, by stimulating TrkB in the PFC, CA3 and DG. Pretreatment with Nrf2 activator sulforaphane (SFN) prevented the depression-like phenotype induced after repeated social defeat stress. Interestingly, dietary intake of 0.1% glucoraphanin (a precursor of SFN) containing food during juvenile and adolescent stages also prevented the depression-like phenotype evoked in adulthood, after repeated social defeat stress. These findings suggest that Keap1-Nrf2 system plays a key role in depression and that dietary intake of SFN-rich food during juvenile stages and adolescence can confer stress resilience in adulthood.

Depression is one of the most common psychiatric disorders in the world. The World Health Organization estimates that more than 350 million individuals of all ages suffer from depression, highlighting this illness as a major contributor to the global burden of disease^1^,^2^. For this reason, new therapeutic approaches are needed to prevent or delay the progression of depression. Over the past decade, there has been increasing interest in the potential benefits of early intervention for psychiatric disorders. Accumulating evidence suggests that nutrition has a high impact on the development of depression^3^,^4^,^5^,^6^,^7^. Recent meta-analyses demonstrated that high intake of fruit, vegetables, fish, and whole grains are associated with a reduced risk of depression^8^,^9^. Neurodevelopment in early adolescence is a key stage during maturation, characterized by various structural, neurochemical, and molecular changes in response to genetic and environmental cues. The formation of new neuronal connections during this developmental window also confers a high level of vulnerability to pathologic insults, ranging from stress to dietary deficiencies^10^,^11^. Taken together, nutritional status during early adolescence has a great impact on the onset and severity of psychiatric diseases in adulthood^11^.

Although the precise mechanisms underlying the pathophysiology of depression are unknown, several lines of evidence implicate inflammatory processes in the development of depression^12^,^13^,^14^,^15^,^16^,^17^. Previous reports including meta-analysis demonstrated higher levels of pro-inflammatory cytokines in the blood of drug-free depressed patients compared to healthy controls^18^,^19^,^20^. Some studies using postmortem brain samples showed elevated gene expression of pro-inflammatory cytokines in patients with a history of depression^21^,^22^. Taken together, it is likely that both peripheral and central inflammation plays a crucial role in the pathophysiology of depression.

Nuclear factor (erythroid 2-derived)-like 2 (Nrf2) is a transcription factor with a central role in cellular defense against oxidative and electrophilic insults^23^,^24^,^25^,^26^. It binds to antioxidant response elements (ARE) located in the promoter region of genes encoding many phase II detoxifying or antioxidant enzymes and related stress-responsive proteins^23^,^24^,^25^,^26^. Under normal conditions, Nrf2 is repressed by Keap1 (Kelch-like erythroid cell-derived protein with CNC homology [ECH]-associated protein 1), which is an adaptor protein for the degradation of Nrf2^25,26^. During oxidative stress, Nrf2 is de-repressed and activates the transcription of cytoprotective genes^25^,^26^. Additionally, the Keap1-Nrf2 system is also involved in attenuating inflammation-associated pathogenesis^24^,^25^,^26^,^27^,^28^.

The potent anti-inflammatory and naturally occurring compound sulforaphane (SFN: 1-isothiocyanato-4-methylsulfinylbutane) is an organosulfur compound derived from a glucosinolate precursor glucoraphanin (GF: a glucosinolate, or β-thioglucoside-N-hydroxysulfate) found in cruciferous vegetables, such as broccoli sprout^29^,^30^,^31^,^32^,^33^,^34^. It is well known that GF can be converted to SFN by the endogenous enzyme, myrosinase^34^. The protection afforded by SFN is thought to be mediated via activation of the Nrf2 pathway with subsequent up-regulation of phase II detoxification enzymes and antioxidant proteins, through an enhancer sequence referred to as the electrophilic-responsive element or ARE^25^,^26^.

The purpose of this study was to examine the role of Keap1-Nrf2 signaling in the pathophysiology of depression using the potent Nrf2 activator, SFN and *Nrf2* knock-out (KO) mice. In addition, we examined the role of brain-derived neurotrophic factor (BDNF) and its receptor tropomyosin-receptor-kinase B (TrkB) signaling in selected brain regions, since BDNF-TrkB signaling is integral to the pathophysiology of depression^35^,^36^,^37^,^38^,^39^,^40^,^41^,^42^. Finally, we examined whether dietary intake of 0.1% GF food during the juvenile and adolescence can prevent depression-like behaviors by repeated social defeat stress.

## Results

### Role of Nrf2 in SFN-induced potentiation of NGF-induced neurite outgrowth

**Figure 1:**
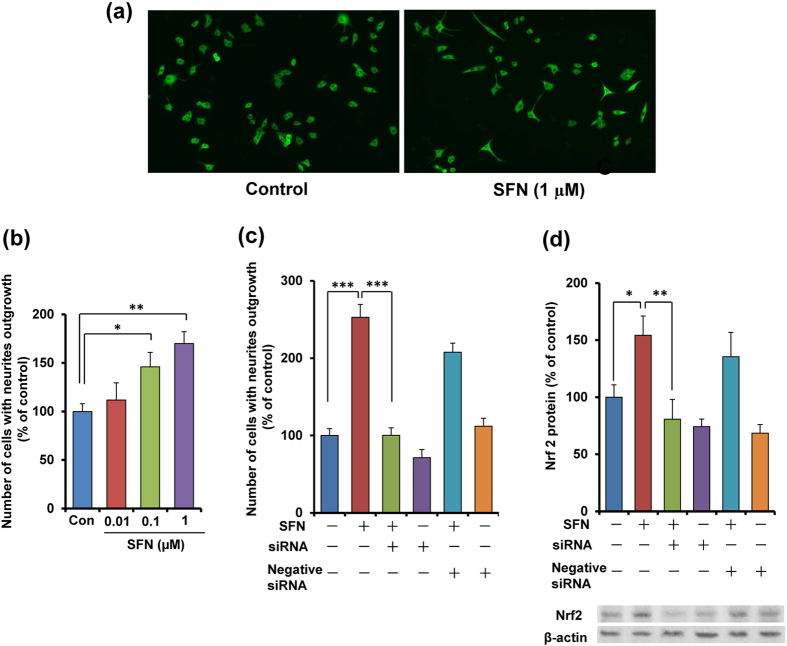
Potentiation of neurite outgrowth by SFN. (**a**) Representative photographs of microtubule-associated protein 2 (MAP-2) immunocytochemistry in PC12 cells. Control: NGF (2.5 ng ml^−1^) alone, SFN: NGF (2.5 ng ml^−1^) + SFN (1.0 μM). (**b**) Effects of SFN on NGF-induced neurite outgrowth in PC12 cells. SFN (0.01, 0.1 or 1.0 μM) potentiated NGF-induced neurite outgrowth in PC12 cells, in a concentration-dependent manner. All data represent the mean ± S.E.M. (n = 6–12) *P < 0.05, ***P < 0.001 as compared with the control group (one-way ANOVA). (**c**) The potentiating effects of SFN (1.0 μM) on NGF-induced neurite outgrowth were significantly antagonized by treatment with *Nrf2* siRNA, but not negative siRNA. Neither *Nrf2* siRNA nor negative siRNA alone altered NGF (2.5 ng ml^−1^)-induced neurite outgrowth. All data represent the mean ± S.E.M. (n = 8). ***P < 0.001 as compared with the SFN (1.0 μM) group (one-way ANOVA). (**d**) The potentiating effects of SFN (1.0 μM) on Nrf2 protein levels were significantly antagonized by treatment with *Nrf2* siRNA, but not the negative siRNA. In contrast, neither *Nrf2* siRNA nor negative siRNA alone altered levels of Nrf2 protein in the control (NGF (2.5 ng ml^−1^)-treated) group. All data represent the mean ± S.E.M. (n = 4). *P < 0.05, **P < 0.01 as compared with the SFN (1.0 μM) group (one-way ANOVA).

Since the current antidepressants are known to affect the neuronal plasticity, we examined the effects of SFN on nerve growth factor (NGF)-induced neurite outgrowth in PC12 cells. MAP-2 (microtubule associated protein-2) immunocytochemistry showed that SFN increased the number of cell with neurite outgrowth in PC12 cells (Fig. 1a). SFN (0.01, 0.1 or 1.0 μM) significantly (one-way ANOVA: F_3,38_ = 7.010, P = 0.001) increased the number of cells with neurite outgrowth in PC12 cells, in a concentration-dependent manner (Fig. 1b).

To investigate whether Nrf2 plays a role in SFN’s potentiation of NGF-induced neurite outgrowth, we examined the effect of *Nrf2* gene knock-down. One-way ANOVA of neurite outgrowth data revealed significant effects (F_5,47_ = 38.56, P < 0.001), the potentiating effects of SFN (1.0 μM) on NGF-induced neurite outgrowth were significantly attenuated by treatment with *Nrf2* siRNA, but not the negative control (Fig. 1c). In contrast, treatment with *Nrf2* siRNA or the negative control alone did not alter NGF-induced neurite outgrowth in PC12 cells (Fig. 1c). Furthermore, SFN (1.0 μM) significantly increased Nrf2 protein in PC12 cells (Fig. 1d). One-way ANOVA revealed significant effects (F_5,23_ = 10.47, P < 0.001). The *Nrf2* siRNA, but not negative control of siRNA, significantly blocked SFN-induced Nrf2 protein expression. In the absence of SFN, *Nrf2* siRNA or negative control did not alter basal levels of Nrf2 protein (Fig. 1d). These findings suggest that SFN can potentiate NGF-induced neurite outgrowth via activation of Nrf2.

### Reduction of Keap1-Nrf2 signaling in the brain from social defeat stress model of depression

**Figure 2:**
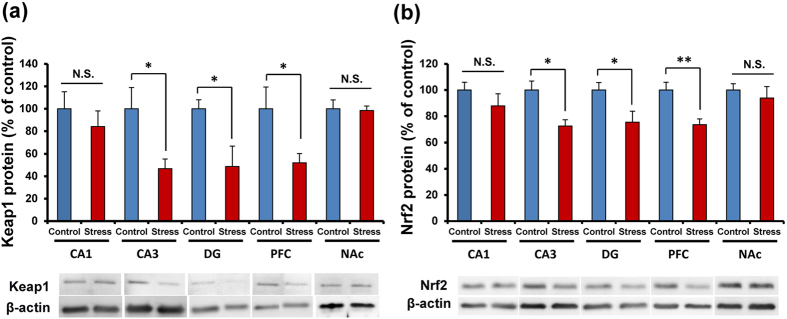
Protein levels of Keap1 and Nrf2 in the brain from mice with depression-like phenotype. (**a**,**b**) The levels of Keap1 and Nrf2 proteins in the mouse brain regions from social defeat stress (susceptible) mice and control mice (n = 5 or 6). Data represent the mean ± S.E.M. *P < 0.05 and **P < 0.01 compared with the control group (Student t-test).

We performed Western blot analysis of Keap1 and Nrf2 proteins in the brain regions (CA1, CA3, dentate gyrus (DG) of hippocampus, prefrontal cortex (PFC), nucleus accumbens (NAc)) of susceptible mice by social defeat stress model. Protein levels of Keap1 and Nrf2 in the CA3, DG, and PFC from mice with depression-like phenotype were significantly (CA3: Keap1, P = 0.037, Nrf2, P = 0.011, DG: Keap1, P = 0.029, Nrf2, P = 0.040, PFC: Keap1, P = 0.045, Nrf2, P = 0.005) lower than those of control mice (Fig. 2a,b). In contrast, protein levels of Keap1 and Nrf2 in the CA1 and NAc were not different (Fig. 2a,b). These findings suggest that decreased levels of Keap1 and Nrf2 in the CA3, DG, and PFC may be implicated in the pathophysiology of depression after social defeat stress.

### Depression-like phenotypes, altered BDNF-TrkB signaling and inflammation in *Nrf2* KO mice

**Figure 3:**
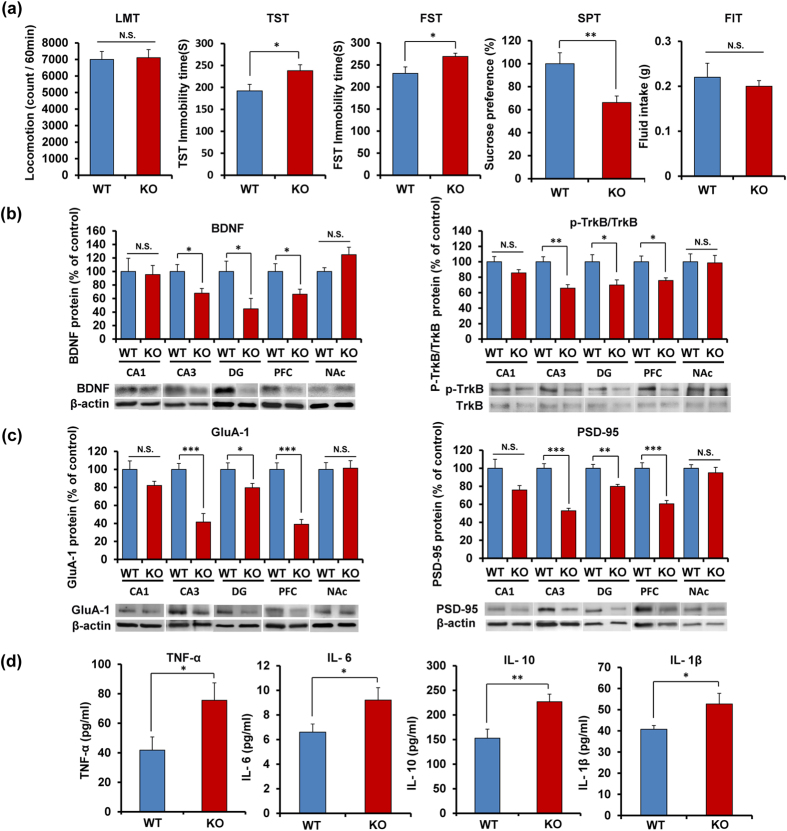
Depression-like phenotypes of *Nrf2* KO mice and inflammation in *Nrf2* KO mice. (**a**) The behavior tests in WT and *Nrf2* KO mice. Locomotion (LMT); tail-suspension test (TST); forced swimming test (FST), 1% sucrose preference test (SPT) and total fluid intake test (FIT) for SPT. Data represent the mean ± S.E.M (n = 9 or 10). *P < 0.05, **P < 0.01 compared with the WT group (Student t-test). (**b**,**c**) Western blot of BDNF, p-TrkB/TrkB, GluA-1 and PSD-95 in WT and *Nrf2* KO mice. Data represent the mean ± S.E.M (n= 6 or 7). *P < 0.05, **P < 0.01, ***P < 0.001 compared with the WT group (Student t-test). (**d**) Serum levels of TNF-α, IL-6, IL-10 and IL-1β in WT and *Nrf2* KO mice. Data represent the mean ± S.E.M (n = 9–12). *P < 0.05, **P < 0.01 compared with the WT group (Student t-test).

Locomotion showed no difference (P = 0.702) between the two groups (Fig. 3a). In the tail-suspension test (TST) and forced swimming test (FST), the immobility times of *Nrf2* KO mice were significantly (TST: P = 0.034; FST: P = 0.017) higher than those of wild-type (WT) mice (Fig. 3a). In the 1% sucrose preference test (SPT), the sucrose preference of *Nrf2* KO mice was significantly (P = 0.009) lower than that of WT mice (Fig. 3a). In addition, the total fluid intake (FIT) for SPT in WT mice and *Nrf2* KO mice was not different (Fig. 3a). These findings show that *Nrf2* KO mice have depression-like phenotype including anhedonia.

Since BDNF-TrkB signaling plays a key role in depression-like phenotype in rodents^35^,^36^,^37^,^38^,^39^,^40^,^41^,^42^, we examined the role of BDNF-TrkB signaling in the selected brain regions of *Nrf2* KO mice. Western blot analyses of BDNF, TrkB, and phosphorylated TrkB (p-TrkB) in the selected brain regions (CA1, CA3, DG, PFC, NAc) from WT mice and *Nrf2* KO mice were performed. Protein levels of BDNF in the CA3, DG and PFC of *Nrf2* KO mice were significantly (CA3: P = 0.024, DG: P = 0.029, PFC: P = 0.041) lower than those of WT mice. Furthermore, the ratio of p-TrkB to total TrkB in the CA3, DG, and PFC of KO mice was also significantly (CA3: P = 0.004, DG: P = 0.036, PFC: P = 0.031) lower than that of WT mice (Fig. 3b). In contrast, the levels of BDNF and the ratio of p-TrkB to total TrkB in the CA1 and NAc remained the same (Fig. 3b).

Next, we measured the markers (AMPA receptor 1 (GluA1), and postsynaptic density protein 95 (PSD-95)) for synaptogenesis. Protein levels of GluA1 and PSD-95 in the CA3, DG, and PFC of KO mice were significantly (CA3: GluA1, P < 0.001, PSD-95, P < 0.001, DG: GluA1, P = 0.041, PSD-95, P = 0.001, PFC: GluA1, P < 0.001; PSD-95: P < 0.001) lower than those of WT mice, although levels of GluA1 and PSD-95 in the CA1 and NAc were unaltered (Fig. 3c).

Furthermore, we measured the serum levels of pro-inflammatory cytokines, including tumor necrosis factor (TNF)-α, interlukin-6 (IL-6), interlukin-10 (IL-10) and interlukin-1β (IL-1β) in WT mice and *Nrf2* KO mice. Serum levels of TNF-α (P = 0.046), IL-6 (P = 0.038), IL-10 (P = 0.005) and IL-1β (P = 0.040) in the *Nrf2* KO mice were significantly higher than those of WT mice, suggesting that *Nrf2* KO mice have inflammation (Fig. 3d).

### Antidepressant effect of TrkB agonist 7,8-dihydroxyflavone (7,8-DHF) in *Nrf2* KO mice

**Figure 4:**
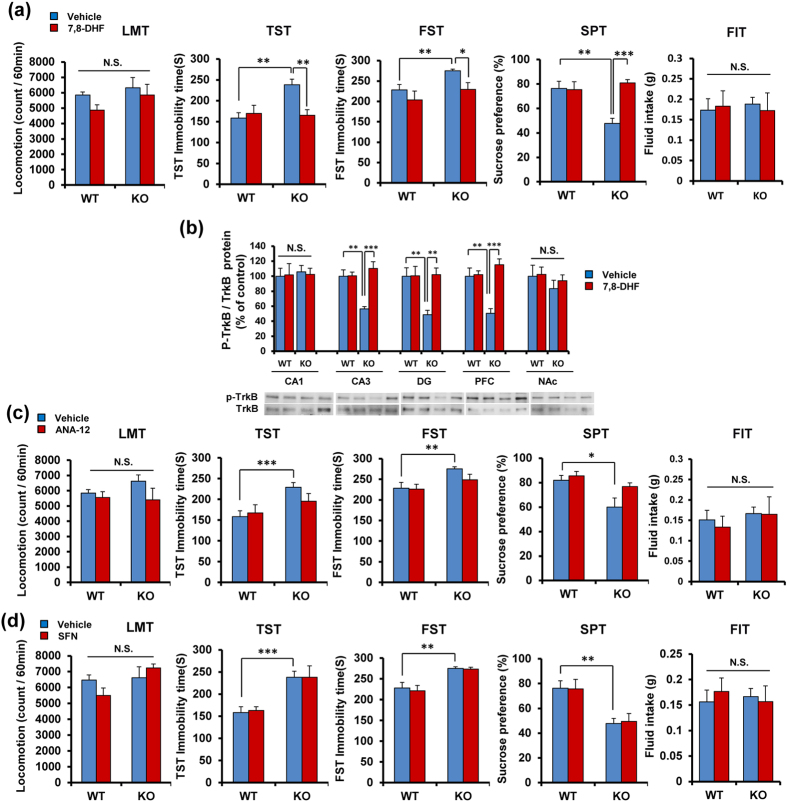
Antidepressant effect of 7,8-DHF, a TrkB agonist, in *Nrf2* KO mice. (**a**) 7,8-DHF (10 mg/kg) or vehicle (10 ml/kg) was administered i.p. into WT and *Nrf2* KO mice. Behavioral tests were performed 1 hour after injection. Locomotion (LMT); tail-suspension test (TST); forced swimming test (FST), 1% sucrose preference test (SPT) and total fluid intake test (FIT) for SPT. Data represent the mean ± S.E.M. (n = 9–11). *P < 0.05, **P < 0.01, ***P < 0.001 compared with vehicle-treated KO group (two-way ANOVA). (**b**) Levels of p-TrkB/TrkB ratio in the mouse brain regions after injection of 7,8-DHF. Data represent the mean ± S.E.M (n = 5 or 6). **P < 0.01, ***P < 0.001 compared with vehicle-treated KO group (two-way ANOVA). (**c**) ANA-12 did not show antidepressant effect in the *Nrf2* KO mice. ANA-12 (0.5 mg/kg) or vehicle (10 ml/kg) was administered i.p. into WT and *Nrf2* KO mice. Behavioral tests were performed 1 hour after injection. Locomotion (LMT); tail-suspension test (TST); forced swimming test (FST); 1% sucrose preference test (SPT) and total fluid intake test (FIT) for SPT. Data represent the mean ± S.E.M. (n = 8–11). *P < 0.05, **P < 0.01, ***P < 0.001 compared with vehicle-treated KO group (two-way ANOVA). (**d**) SFN did not show antidepressant effect in the *Nrf2* KO mice. SFN (10 mg/kg) or vehicle (10 ml/kg) was administered i.p. into WT and *Nrf2* KO mice. Behavioral tests were performed 1 hour after injection. Locomotion (LMT); tail-suspension test (TST); forced swimming test (FST), 1% sucrose preference test (SPT) and total fluid intake test (FIT) for SPT. Data represent the mean ± S.E.M. (n = 10–11). **P < 0.01, ***P < 0.001 compared with vehicle-treated KO group (two-way ANOVA).

Since *Nrf2* KO mice showed decreased BDNF-TrkB signaling in the CA3, DG and PFC, we examined whether the TrkB agonist 7,8-DHF^43^,^44^,^45^,^46^,^47^,^48^ shows antidepressant effects in depression-like phenotype of *Nrf2* KO mice. A single dose of 7,8-DHF (10 mg/kg) had no effect on locomotion (Fig. 4a). In the TST and FST, 7,8-DHF (10 mg/kg) significantly attenuated the increased immobility time of *Nrf2* KO mice. In the TST, two-way ANOVA revealed significant effects (genotype: F_1,39_ = 7.134, P = 0.011, treatment: F_1,39_ = 4.912, P = 0.033, interaction: F_1,39_ = 7.679, P = 0.009) (Fig. 4a). In the FST, two-way ANOVA revealed significant effects (genotype: F_1,39_ = 5.756, P = 0.022, treatment: F_1,39_ = 5.434, P = 0.026, interaction: F_1,39_ = 0.495, P = 0.486) (Fig. 4a). Furthermore, 7,8-DHF (10 mg/kg) significantly attenuated the decreased sucrose preference of *Nrf2* KO mice. Two-way ANOVA revealed significant effects (genotype: F_1,40_ = 5.194, P = 0.029, treatment: F_1,40_ = 10.25, P = 0.003, interaction: F_1,40_ = 11.41, P = 0.002) (Fig. 4a). In addition, the total FIT for SPT among four groups was not different (Fig. 4a). These findings suggest that a TrkB agonist 7,8-DHF showed antidepressant effects in the *Nrf2* KO mice.

A single administration of 7,8-DHF (10 mg/kg) significantly attenuated the decreased ratio of p-TrkB to total TrkB in the CA3, DG and PFC of *Nrf2* KO mice. Two-way ANOVA revealed significant effects (CA3, genotype: F_1,21_ = 5.512, P = 0.031, treatment: F_1,21_ = 14.02, P = 0.001, interaction: F_1,21_ = 13.44, P = 0.002; DG, genotype: F_1,21_ = 5.461, P = 0.031, treatment: F_1,21_ = 6.425, P = 0.021, interaction: F_1,21_ = 6.200, P = 0.023, PFC: genotype: F_1,21_ = 4.948, P = 0.039, treatment: F_1,21_ = 16.53, P = 0.001, interaction: F_1,21_ = 14.43, P = 0.001) (Fig. 4b). However, there were no changes in the CA1 and NAc (Fig. 4b).

In contrast, a single administration of TrkB antagonist ANA-12 (0.5 mg/kg)^45^,^46^,^47^,^48^,^49^ did not affect depression-like phenotype in *Nrf2* KO mice (Fig. 4c), suggesting a role of TrkB signaling in the PFC and hippocampus for depression-like phenotype. In addition, a single administration of SFN (10 mg/kg)^50^,^51^,^52^ did not affect depression-like phenotype in *Nrf2* KO mice (Fig. 4d), suggesting a role of Nrf2 in depression-like phenotype.

### Pretreatment with SFN prevented the onset of depression-like behavior by repeated social defeat stress

**Figure 5:**
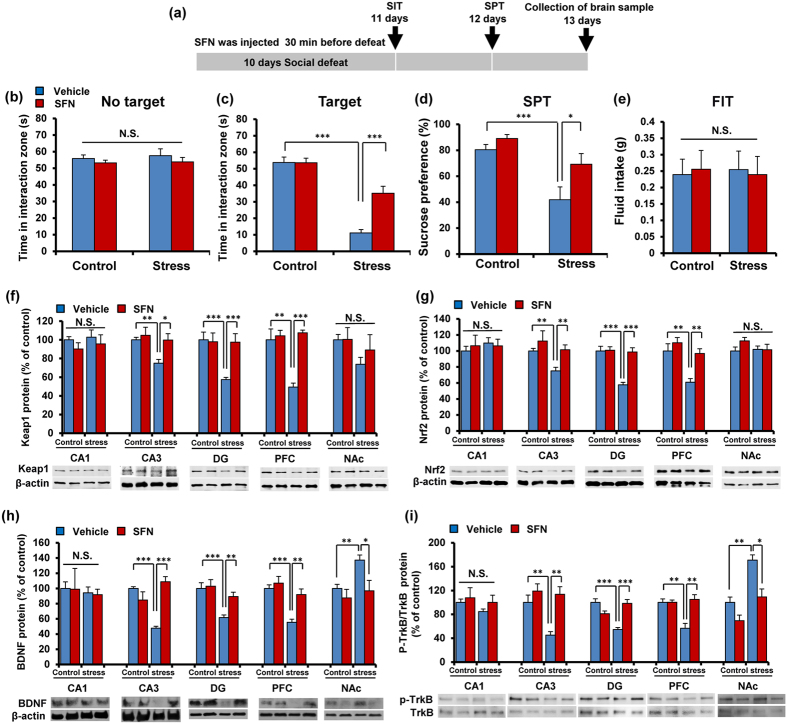
Pretreatment with SFN prevents depression after repeated social defeat stress. (**a**) The schedule of treatment and behavioral evaluations. (**b**) Social interaction test (SIT): no target, (**c**) SIT: target. (**d**) 1% sucrose preference test (SPT), (**e**) total fluid intake test (FIT) for SPT. Data represent the mean ± S.E.M. (n = 10–14). *P < 0.05, ***P < 0.001 compared with the vehicle-treated stress group (two-way ANOVA). (**f**,**g**) Western blot of Keap1, Nrf2 in the mouse brain regions. Data represent the mean ± S.E.M. (n = 5 or 6). *P < 0.01, **P < 0.01, ***P < 0.001 compared with the vehicle-treated stressed group (two-way ANOVA). (**h**,**i**) Western blot of BDNF, p-TrkB/TrkB in the mouse brain regions. Data represent the mean ± S.E.M. (n = 5 or 6). *P < 0.01, **P < 0.01, ***P < 0.001 compared with the vehicle-treated stress group (two-way ANOVA).

The aforementioned findings suggest that Nrf2 plays a key role in pathophysiology of depression. In this study, we examined whether pretreatment with SFN could prevent depression-like phenotype by repeated social defeat stress (Fig. 5a). In the social interaction test (no target), the social interaction time has no significant changes in all groups (Fig. 5b). In the social interaction test (target), SFN significantly attenuated the decreased social avoidance time in stressed mice (Fig. 5c). Two-way ANOVA on the data (target) revealed significant effects (stress: F_1,47_ = 88.28, P < 0.001, SFN: F_1,47_ = 13.56, P = 0.001, interaction: F_1,47_ = 13.89, P = 0.001) (Fig. 5c). In the SPT, SFN significantly attenuated the decreased sucrose preference of stressed mice (Fig. 5d). Two-way ANOVA revealed significant effects (stress: F_1,50_ = 24.20, P < 0.001, SFN: F_1,50_ = 9.238, P = 0.004, interaction: F_1,50_ = 2.478, P = 0.122) (Fig. 5d). In addition, the total FIT for SPT among four groups was not different (Fig. 5e).

Next, we performed Western blot analyses of Keap1, Nrf2, BDNF, TrkB, and p-TrkB in the selected regions. We found that pretreatment with SFN significantly attenuated the decreased levels of Keap1 and Nrf2 in the CA3, DG, and PFC of stressed mice (Fig. 5f,g). Two-way ANOVA of Keap1 and Nrf2 data revealed significant effects (Keap1: CA3, stress: F_1,21_ = 5.332, P = 0.033, treatment: F_1,21_ = 5.161, P = 0.036, interaction: F_1,21_ = 2.335, P = 0.144; DG, stress: F_1,21_ = 7.190, P = 0.015, treatment: F_1,21_ = 5.617, P = 0.029, interaction: F_1,21_ = 6.914, P = 0.017; PFC, stress: F_1,21_ = 9.795, P = 0.006, treatment: F_1,21_ = 16.948, P = 0.001, interaction: F_1,21_ = 12.540, P = 0.002; Nrf2: CA3, stress: F_1,21_ = 4.804, P = 0.042, treatment: F_1,21_ = 5.653, P = 0.029, interaction: F_1,21_ = 0.739, P = 0.401; DG, stress: F_1,21_ = 19.848, P < 0.001, treatment: F_1,21_ = 17.549, P = 0.001, interaction: F_1,21_ = 16.206, P = 0.001; PFC, stress: F_1,21_ = 14.039, P = 0.001, treatment: F_1,21_ = 10.845, P = 0.004, interaction: F_1,21_ = 3.363, P = 0.083) (Fig. 5f,g).

Pretreatment with SFN significantly attenuated the decreased levels of BDNF in the CA3, DG, and PFC from stressed mice. In contrast, pretreatment with SFN significantly attenuated the increased levels of BDNF in the NAc from stressed mice (Fig. 5h). Two-way ANOVA of BDNF data revealed significant effects (CA3, stress: F_1,21_ = 4.653, P = 0.045, treatment: F_1,21_ = 12.28, P = 0.003, interaction: F_1,21_ = 34.12, P < 0.001; DG, stress: F_1,21_ = 14.37, P = 0.001, treatment: F_1,21_ = 4.998, P = 0.038, interaction: F_1,21_ = 3.277, P = 0.087; PFC, stress: F_1,21_ = 18.86, P < 0.001, treatment: F_1,21_ = 10.06, P = 0.005, interaction: F_1,21_ = 4.587, P = 0.046; NAc, stress: F_1,21_ = 5.388, P = 0.032, treatment: F_1,21_ = 6.961, P = 0.017, interaction: F_1,21_ = 1.958, P = 0.179) (Fig. 5h).

To clarify whether TrkB activation or inhibition underpins depression-like phenotype in stressed (susceptible) mice, we performed the immunoblot blot analyses of TrkB and phosphorylated TrkB (p-TrkB), an activated form of TrkB in the brain regions. Stressed mice showed decreased levels of p-TrkB in the CA3, DG, and PFC as well as increased levels of p-TrkB in the NAc. In contrast, the levels of total TrkB protein were not altered among the four groups. Pretreatment with SFN significantly attenuated the decreased ratio of p-TrkB to total TrkB in CA3, DG and PFC of stressed mice (Fig. 5i). In contrast, pretreatment with SFN significantly attenuated the increased levels of p-TrkB to total TrkB in NAc from stressed mice (Fig. 5i). Two-way ANOVA of p-TrkB/TrkB ratio revealed significant effects (CA3, stress: F_1,20_ = 6.296, P = 0.023, treatment: F_1,20_ = 13.243, P = 0.002, interaction: F_1,20_ = 4.205, P = 0.056; DG, stress: F_1,20_ = 5.868, P = 0.027, treatment: F_1,20_ = 4.638, P = 0.046, interaction: F_1,20_ = 29.69, P < 0.001; PFC, stress: F_1,21_ = 7.379, P = 0.014, treatment: F_1,21_ = 11.79, P = 0.003, interaction: F_1,21_ = 11.53, P = 0.003; NAc, stress: F_1,21_ = 25.65, P < 0.001, treatment: F_1,21_ = 17.77, P = 0.001, interaction: F_1,21_ = 2.017, P = 0.173) (Fig. 5i).

### Dietary intake of 0.1% glucoraphanin during the juvenile and adolescence can prevent depression-like behaviors by repeated social defeat stress

**Figure 6:**
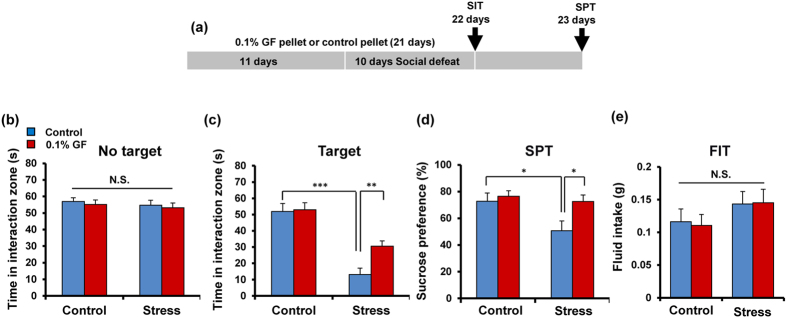
Dietary intake of 0.1% GP during the juvenile and adolescence prevents depression after repeated social defeat stress. (**a**) The schedule of dietary intake of 0.1% glucoraphanin (GP) and behavioral evaluations. (**b**) Social interaction test (SIT): no target, (**c**) SIT: target, (**d**) 1% sucrose preference test (SPT), (**e**) total fluid intake test (FIT) for SPT. Data represent the mean ± S.E.M. (n = 16 or 17). *P < 0.05, **P < 0.01, ***P < 0.001 compared with the vehicle-treated stressed group (two-way ANOVA).

Nutritional status during juvenile and adolescence has a great impact on the onset and severity of psychiatric diseases in adulthood^10^,^11^. Mice (5-weeks old) were received dietary intake of 0.1% GF containing food pellets for 21 days. Subsequently, repeated social defeat stress was performed for 10 days (Fig. 6a). Pellet including 0.1% GF was prepared as reported previously^52^. In the social interaction test (no target), the social interaction time for all groups was not different (Fig. 6b). In the social interaction test (target), mice with pellets including 0.1% GF significantly attenuated the decreased social avoidance time in stressed mice (Fig. 6c). Two-way ANOVA revealed significant effects (stress: F_1,64_ = 54.90, P < 0.001, treatment: F_1,64_ = 5.011, P = 0.029, interaction: F_1,64_ = 3.927, P = 0.052) (Fig. 6c). In the SPT, treatment with pellets including 0.1% GF significantly attenuated the decreased sucrose preference of stressed mice. Two-way ANOVA analysis of SPT data revealed significant effects (stress: F_1,64_ = 5.844, P = 0.019, treatment: F_1,64_ = 4.382, P = 0.040, interaction: F_1,64_ = 2.971, P = 0.090) (Fig. 6d). In addition, the total FIT for SPT among the four groups was not different (Fig. 6e). These findings suggest that dietary intake of 0.1% GF can prevent the onset of depression-like phenotype by repeated social defeat stress.

## Discussion

The present data demonstrated a key role for the Keap1-Nrf2 system in the pathophysiology of depression. The major findings of this study were: First, through Nrf2 activation, SFN can potentiate NGF-induced neurite outgrowth in PC12 cells, indicating that Nrf2 activators such as SFN are capable of enhancing the neuronal plasticity. Secondly, Nrf2 and Keap1 proteins showed decreased expression in the CA3, DG, and PFC of mice with depressed-like phenotype after repeated social defeat stress. Thirdly, *Nrf2* KO mice displayed a depression-like phenotype including anhedonia, while the TrkB agonist 7,8-DHF promoted an antidepressant effect in *Nrf2* KO mice. However, neither the TrkB antagonist ANA-12 nor the Nrf2 activator SFN had an effect on the depression-like phenotype seen in *Nrf2* KO mice. It is likely that decreased BDNF-TrkB signaling and synaptogenesis in the CA3, DG, and PFC of *Nrf2* KO mice promotes the depression-like phenotype of these KO mice. Moreover, serum levels of pro-inflammatory cytokines in *Nrf2* KO mice were higher than those of WT mice, suggesting that *Nrf2* KO mice show enhanced inflammation. Lastly, pretreatment with SFN significantly attenuated the decreased social avoidance time and sucrose preference in repeated social defeat stress mice. Interestingly, dietary intake of 0.1% GF pellets during juvenile and adolescent stages significantly attenuated the decreased social avoidance time and sucrose preference in repeated social defeat stress mice during adulthood. All these findings implicate the Keap1-Nrf2 system in the pathophysiology of depression, as well as suggesting that the dietary intake of 0.1% GF pellets during juvenile and adolescent development may confer stress resilience in adulthood.

A microarray study showed that loss of *Nrf2* gene altered the expression of various categories of genes associated with metabolism, channel or transport activity, receptor-mediated signal transduction, cell adhesion and transcriptional regulation^53^. Interestingly, the expression levels of detoxification enzyme genes, such as sulfotransferase 1B1 (*Sult1b1*), glutathione-S-transferase (GST) M1 (*Gstm1*), GSTM3 (*Gstm3*) and aldehyde oxidase 1 (*Aox1*), were decreased in the brain regions of *Nrf2* KO mice^53^. Given the role of oxidative stress in depression^54^,^55^, it is likely that decreased levels of these genes in the brain may be involved in the depression-like phenotype of *Nrf2* KO mice although a further detailed study is needed. In this study, we found that social defeat stress significantly decreased the levels of Nrf2 and Keap1 protein in the hippocampus and PFC. Moreover, SFN could attenuate the decreased levels of Nrf2 and Keap1 proteins in the hippocampus and PFC of stressed mice. These results suggest that Keap1-Nrf2 signaling may play a role in the pathophysiology of depression, and that SFN is prophylactic compound which can stimulate Keap1-Nrf2 signaling pathway.

BDNF-TrkB signaling plays a key role in the pathophysiology of depression^35^,^36^,^37^,^38^,^39^,^40^,^41^,^42^. Studies using postmortem brain samples recorded reduced levels of BDNF in the hippocampus and PFC of psychiatric disorder patients who had committed suicide, compared with non-psychiatric controls^56^,^57^. Recently, we reported that BDNF levels in hippocampus and PFC of mice with depression-like phenotype were lower than control mice, conversely, BDNF levels in the NAc of mice with depression-like phenotype were higher than control mice^47^,^48^,^58^,^59^. A single infusion of BDNF or the TrkB agonist, 7,8-DHF into the DG and CA3 of the hippocampus promoted rapid and long-lasting antidepressant effects in learned helplessness models of depression^60^,^61^, whereas a single infusion of ANA-12 into the NAc showed antidepressant effects^61^. The loss of BDNF in the forebrain attenuated the action of antidepressants^62^, and responses elicited by antidepressants were lost in mice with either reduced brain BDNF levels or inhibited TrkB signaling^63^,^64^. These results suggest that abnormal BDNF levels in the hippocampus and PFC, and the NAc play a causative role in the pathophysiology of depression. In this study, we detected decreased BDNF-TrkB signaling in the CA3, DG and PFC of *Nrf2* KO mice. We also found that the TrkB agonist, 7,8-DHF conferred an antidepressant effect, by stimulating TrkB in these regions. Thus, it is likely that decreased BDNF-TrkB signaling in CA3, DG and PFC regions, precipitated by a deletion of the *Nrf2* gene, may mediate depression-like phenotypes in *Nrf2* KO mice. Previous reports also showed that *Nrf2* KO mice show depression-like phenotype^65^, and that BDNF levels in the cortex and hippocampus of *Nrf2* KO mice were lower than control mice^66^. The precise mechanisms underlying decreased BDNF levels in the PFC and hippocampus of *Nrf2* KO mice are currently unknown. Further detailed studies on the role of Nrf2 in BDNF-TrkB signaling in the brain are needed. Previously, we reported that TrkB antagonist ANA-12 showed antidepressant effect by inhibiting TrkB signaling pathway in the NAc of mice with depression-like phenotye^47^,^48^. In this study, TrkB antagonist ANA-12 did not show antidepressant effect since the BDNF-TrkB signaling was not increased in the NAc of *Nrf2* KO mice.

Growing evidence suggests that inflammatory processes play a role in the pathophysiology of depression^12^,^13^,^14^,^15^,^16^,^17^. In this study, we found increased serum levels of pro-inflammatory cytokines, such as TNF-α, IL-6, IL-10 and IL-1β, in *Nrf2* KO mice, indicating increased inflammation in these mice. Thus, it seems that abnormal pro-inflammatory cytokine levels in the serum of *Nrf2* KO mice may play a causative role in the pathophysiology of depression, since higher levels of these cytokines were detected in the blood of patients with depression^67^,^68^. Recently, we reported that a single administration of lipopolysaccharide (LPS) induced higher serum levels of these cytokines and a depression-like phenotype in mice^47^, suggesting an inflammation model of depression. Given the role of Keap1-Nrf2 signaling in inflammation, the depression-like phenotype of *Nrf2* KO mice is noteworthy, consistent with previous report showing that *Nrf2* KO mice exhibit an increase of inflammatory markers^65^. Although the reasons underlying inflammation in *Nrf2* KO mice are currently unknown, it seems that microgliosis due to the lack of Nrf2 may lead to activation of NF-κB, AP-1 and their up-regulating kinases which, in turn, increase the production of pro-inflammatory cytokines^65^.

Nutritional status during early adolescence has a great impact on the onset and severity of psychiatric diseases in adulthood^10^,^11^. Over the past decade, there has been increasing interest in the potential benefits of early intervention for psychiatric diseases^10^,^11^. Therefore, it is of great interest to study whether dietary intake of 0.1% GF during juvenile and adolescence phases can prevent the depression-like behavior in adulthood, after repeated social defeat stress. Here, we found that dietary intake of 0.1% GF during juvenile and adolescence phases was capable of preventing depression-like behavior in adulthood, after repeated social defeat stress. A recent randomized, double-blinded, placebo-controlled study demonstrated that treatment with SFN-rich broccoli sprout extract significantly improved social interaction, abnormal behavior and verbal communication in young men with autism spectrum disorder^69^. In addition, we reported that supplementation with GF-rich broccoli sprout extract for eight weeks was effective in treating cognitive impairment in medicated patients with schizophrenia^70^. Although the precise mechanisms underlying the prophylactic and therapeutic effects of SFN are currently unknown, dietary intake of SFN-rich broccoli sprout extract may be able to regulate gene expression through epigenetic mechanisms.

There is a limitation of this study. In this study, we used the schedule of dietary intake of 0.1% GF during juvenile and adolescent stages (from 5-weeks old to 8-week old), indicating that GF (or SFN) in the body may affect behavioral effects. Previously, we reported that dietary intake of 0.1% GF during juvenile and adolescent stages (from 4-week old to 8-week old) and subsequent normal pellet (from 8-week old to 10-week old) could prevent phencyclidine-induced cognitive deficits in adulthood^52^. It is, therefore, of great interest to study whether dietary intake of 0.1% GF during juvenile and adolescent stages (from 5-week old to 8-week old) and subsequent normal food from 8-week old and 10-week old can confer stress resilience in adulthood.

In conclusion, our study demonstrated that not only is the Keap1-Nrf2 system crucial to the pathophysiology of depression, but that decreased BDNF-TrkB signaling in the PFC, CA3, and DG plays a role in the depression-like phenotype of *Nrf2* KO mice. Furthermore, dietary intake of 0.1% GF during mouse juvenile and adolescent stages can prevent stress-induced depression-like behavior in adult stages. Therefore, it is possible that dietary intake of GF (or SFN) during childhood and adolescence could prevent the onset of depression in humans during adulthood. In addition, dietary intake of GF (or SFN) may prevent or minimize relapse from remission, induced by inflammation and/or stress in depressed patients.

## Methods and Materials

### Cell culture and quantification of neurite outgrowth

PC12 cells (RIKEN Cell Bank, Tsukuba, Japan) were cultured at 37 °C, 5% CO2 in Dulbecco’s modified Eagle’s medium (DMEM), supplemented with 5% heat-inactivated fetal bovine serum (FBS), 10% heat-inactivated horse serum and 1% penicillin. Medium was changed two to three times a week. PC12 cells were plated onto 24-well tissue culture plates coated with poly-D-lysine/laminin. Cells were plated at relatively low density (0.25 × 10^4^ cells/cm^2^) in DMEM medium containing 0.5% FBS, 1% penicillin-streptomycin. Medium containing a minimal level of serum (0.5% FBS) was used as previously reported^71^. Twenty-four hours after plating, the medium was replaced with DMEM medium containing 0.5% FBS and 1% penicillin-streptomycin with nerve growth factor (NGF: 2.5 ng/ml), with or without SFN (0.01, 0.1 or 1.0 μM).

Four days after incubation with NGF (2.5 ng/ml) with or without specified drugs, morphometric analysis was performed on digitized images of live cells taken under phase contrast illumination, with an inverted microscope linked to a camera. Images of three fields per well were taken, with an average of 100 cells per field. Differentiated cells were counted by visual examination of the field; only cells that had at least one neurite with a length equal to the cell body diameter were counted, and were then expressed as a percentage of the total cells in the field. Counting was performed in a blinded manner. Data were expressed as a percentage of control group (NGF alone).

Cells were fixed for 30 min at room temperature (RT), with 4% paraformaldehyde, permeabilized with 0.2% Triton and then blocked with 5% normal horse serum, in 0.1 M phosphate-buffer saline for 1 h, to reduce nonspecific binding. Cells were incubated overnight at 4 °C with anti-microtubule-associated protein 2 (MAP-2) antibodies (1:1000 dilution in blocking solution, Chemicon International, Temecula, CA, USA). Immunolabeling was visualized with secondary antibodies conjugated to Alexa-488 (1:1000; Invitrogen, Carlsbad, CA, USA). MAP-2 immunocytochemistry was visualized by fluorescence microscopy (Axiovert 200, Carl Zeiss, Oberkochen, Germany).

### Animals

Male adult C57BL/6 mice, aged 8 weeks (body weight 20-25 g, Japan SLC, Inc., Hamamatsu, Japan), CD1 mice, aged 14 weeks (body weight 40–45 g, Japan SLC, Inc., Hamamatsu, Japan) and male adult *Nrf2* homozygous KO mice (*Nrf2*^−/−^) mice^72^ were used in experiments. Male C57BL/6 mice, aged 5 weeks (Japan SLC, Inc., Hamamatsu, Japan) were used in the experiment of dietary intake of 0.1% glucoraphanin (GF) food pellet. Animals were housed under controlled temperature and 12 hour light/dark cycles (lights on between 07:00–19:00), with ad libitum food and water. All experiments were carried out in accordance with the Guide for Animal Experimentation of Chiba University. The protocol was approved by the Chiba University Institutional Animal Care and Use Committee.

### Prepare of 0.1% glucoraphanin (GF) diet

Food pellets (CE-2; Japan CLEA, Ltd., Tokyo, Japan) containing 0.1% glucoraphanin (GF) were prepared as follows. Broccoli sprout extract powder containing SFN precursor GF was industrially produced by KAGOME CO., LTD. In brief, broccoli sprout was grown from specially selected seeds (Brassica Protection Products LLC., Baltimore, MD, USA) for 1 day after the germination. The 1 day broccoli sprout was plunged into boiling water and maintained at 95 °C for 30 minutes, and the sprout residues was removed by filtration. The boiling water extract was mixed with a waxy corn starch dextrin and then spray dried to yield the broccoli sprout extract powder containing 135 mg (approx. 0.31 mmol) of GF per gram. For preparing the animal diet containing 0.1% GF (approx. 2.3 mmol GF per 1 kg-diet), the extract powder was mixed with a basal diet CE-2, and then pelletized at a processing facility (Oriental Yeast Co., ltd., Tokyo, Japan). The GF content in the diet was determined by high performance liquid chromatography as previously described^73^,^74^.

### Drugs and treatment

On the day of injection, fresh solutions were prepared by dissolving compounds in sterile endotoxin-free isotonic saline. (*R,S*)-sulforaphane (SFN; 10 mg/kg, LKT Laboratories, Inc., St Paul, MN, USA) was dissolved in distilled water including 10% corn oil. 7,8-Dihydroxyflavone (7,8-DHF: 10 mg/kg, Catalog number: D1916, Tokyo Chemical Industry, Tokyo, Japan) was prepared in a vehicle of 17% dimethylsulfoxide (DMSO) in phosphate-buffered saline (PBS). ANA-12, N2-(2-{[(2-oxoazepan-3-yl) amino]carbonyl}phenyl)benzo[b] thiophene-2-carboxamide (0.5 mg/kg, Catalog number: BTB06525SC, Maybridge, UK), was dissolved in 1% DMSO in physiological saline. SFN, 7,8-DHF or ANA-12 were administered intraperitoneally (i.p.) into mice. The dose of SFN (10 mg/kg), 7,8-DHF(10 mg/kg) and ANA-12 (0.5 mg/kg) was selected as previously reported^44^,^45^,^46^,^47^,^48^,^49^,^50^.

### Social defeat procedure

The procedure for inducing social defeat stress was performed as previously reported^48^,^59^,^75^. Briefly, C57BL/6 mice were exposed to a different CD1 aggressor mouse each day for 10 min for 10 days. After the social defeat session, the resident CD1 mouse and the intruder mouse were housed in one half of the cage separated by a perforated Plexiglas divider to allow visual, olfactory, and auditory contact for the remainder of the 24-h period. At 24 h after the last session, all mice were housed individually. On day 11, a social avoidance test was performed to identify subgroups of mice that were susceptible and unsusceptible to social defeat stress. Approximately 70% of mice were susceptible in this study. Only susceptible (depressive-like behavior) mice were used in the subsequent experiments.

A social interaction test was performed on one day after the last social defeat session. For this test, an open field arena (42 × 42 cm) was divided into an interaction zone and two opposing corner zones. A mesh-plastic target box (10 × 4.5 cm) was placed into the interaction zone. A test mouse was allowed to roam around the open field arena for 2.5 min with no social target (CD1 mouse) in the mesh box (denoted as ‘no target’ in figures showing results of social-interaction experiments). After this, a novel CD1 mouse was placed in a metal mesh-plastic target box in the interaction zone (denoted as ‘target’ in figures showing results of social-interaction experiments) and the test mouse was placed back into the open arena for another 2.5 min. Using the stopwatch, the amount of time spent in the interaction zone (defined as the 8-cm-wide area surrounding the wiremesh cage) was measured each with or without social target in 2.5 min^76^.

### Behavioral tests

Behavioral tests were performed as reported previously^46^,^47^,^48^,^59^. Locomotion: the mice were placed in experimental cages (length × width × height: 560 × 560 × 330 mm). Locomotor activity of mice was counted by the SCANETMV-40 (MELQUEST Co., Ltd., Toyama, Japan), and cumulative exercise was recorded for 60 minutes. Cages were cleaned between testing session. Tail suspension test (TST): The mice were taken from their home cage and a small piece of adhesive tape was placed approximately 2 cm from the tip of their tail. A single hole was punched in the tape and mice were hung individually, on a hook. The immobility time of each mouse was recorded for 10 minutes. Mice were considered immobile only when they hung passively and completely motionless. Forced swimming test (FST): The mice were placed individually in a cylinder (diameter: 23 cm; height: 31 cm) containing 15 cm of water, maintained at 23 ± 1 °C. Animals were tested in an automated forced-swim apparatus using SCANETMV-40 (MELQUEST Co., Ltd., Toyama, Japan). Immobility time was calculated from activity time as (total) – (active) time, using the apparatus analysis software. Cumulative immobility time was scored for 6 minutes during the test. Sucrose preference test (SPT): Mice were habituated to a 1% sucrose solution for 48 h before the test day. Mice were deprived of water and food for 4 h, followed by a preference test spanning 1 h with water and 1% sucrose, delivered from identical bottles. The bottles containing water and sucrose were weighed before and at the end of this period and the sucrose preference was determined.

### Western blot analysis

The brain samples of CA1, CA3 and DG of the hippocampus, prefrontal cortex (PFC) and NAc from mice were dissected as previously reported^47^,^48^,^59^. Tissue samples or PC12 cells were homogenized in Laemmli lysis buffer. Aliquots (10 μg for mice brain sample and 30 μg for cell sample) of protein were measured using the DC protein assay kit (Bio-Rad, Hercules, CA, USA), and incubated for 5 min at 95 °C, with an equal volume of 125 mM Tris/HCl, pH 6.8, 20% glycerol, 0.1% bromophenol blue, 10% β-mercaptoethanol, 4% sodium dodecyl sulfate, and subjected to sodium dodecyl sulfate polyacrylamide gel electrophoresis, using 10% mini-gels (Mini-PROTEAN TGX Precast Gel; Bio-Rad). Proteins were transferred onto polyvinylidenedifluoride (PVDF) membranes using a Trans Blot Mini Cell (Bio-Rad). For immunodetection, the blots were blocked with 2% BSA in TBST (TBS + 0.1% Tween-20) for 1 h at room temperature (RT), and kept with primary antibodies overnight at 4 °C. The following primary antibody was used: nuclear factor (erythroid 2-derived)-like 2 (Nrf2) (1: 1000, Abcam), Kelch-like erythroid cell-derived protein with CNC homology [ECH]-associated protein 1(Keap1) (1:1000, Abcam) brain derived neurotrophic factor (BDNF) (1:1000, Santa Cruz Biotechnology, Inc., CA), phosphor-TrkB (Tyr 706) (1:200, Santa Cruz Biotechnology, Inc., CA), postsynaptic density protein 95 (PSD-95) (1 μg/ml Invitrogen, Carlsbad, CA), and glutamate receptor 1 (GluA1) (1 μg/ml Abcam, Cambridge, MA). The next day, blot were washed three times in TBST and incubated with horseradish peroxidase conjugated anti-rabbit antibody (1:5000 for Nrf2, BDNF, PSD-95 and GluA-1; 1:1000 for TrkB) and goat anti-rabbit antibody (1:5000 for keap1, 1:2000 for p-TrkB) 1 hour, at RT. After final three washes with TBST, bands were detected using enhanced chemiluminescence (ECL) plus the Western Blotting Detection system (GE Healthcare Bioscience). The blots then were incubated in the stripping buffer (2% SDS, 100 mM β-mercaptoethanol, 62.5 mM Tris-HCl PH 6.8) for 30 min at 60 °C followed by three time washed with TBST. The stripped blots were kept blocking solution for 1 hour and incubated with the primary antibody directed against TrkB (80E3) (1:1000, Cell Signaling Technology, MA) and β-actin. Images were captured with a Fuji LAS3000-mini imaging system (Fujifilm, Tokyo, Japan), and immunoreactive bands were quantified.

### Measurement of pro-inflammatory cytokines

The WT mice and KO mice were anesthetized with pentobarbital, and blood was collected from heart. Blood was centrifuged at 2,000 g for 20 minutes to generate serum samples. The serum samples were diluted 10-fold with ELISA diluent solution (eBioscience, San Diego, CA, USA). The serum levels of tumor necrosis factor (TNF)-α, interlukin-6 (IL-6), interlukin-10 (IL-10) and interlukin-1β (IL-1β) concentrations were measured using a Ready-SET-Go ELISA kit (eBioscience, San Diego, CA, USA) according to the manufacturer’s instructions.

### Statistical Analysis

The data are shown as the mean ± standard error of the mean (S.E.M.). Analysis was performed using PASW Statistics 20 (formerly SPSS statistics; SPSS, Tokyo, Japan). Comparisons between groups were performed using the Student’s t-test, one-way analysis of variance (ANOVA), followed by *post-hoc* Tukey test or two-way ANOVA, when appropriate, *post-hoc* comparisons were performed using the unpaired t-test. The P values of less than 0.05 were considered statistically significant.

## Additional Information

**How to cite this article**: Yao, W. *et al*. Role of Keap1-Nrf2 signaling in depression and dietary intake of glucoraphanin confers stress resilience in mice. *Sci. Rep*. **6**, 30659; doi: 10.1038/srep30659 (2016).
